# In-Host Adaptation of *Salmonella enterica* Serotype Dublin during Prosthetic Hip Joint Infection

**DOI:** 10.3201/eid2412.180214

**Published:** 2018-12

**Authors:** Faten El Sayed, Guillaume Sapriel, Nizar Fawal, Aurelia Gruber, Thomas Bauer, Beate Heym, Caroline Dupont, Henri-Jean Garchon, Jean-Louis Gaillard, Martin Rottman, Simon Le Hello

**Affiliations:** University of Versailles Saint-Quentin, Montigny-le-Bretonneux, France (F. El Sayed, G. Sapriel, B. Heym, H.-J. Garchon, J.-L. Gaillard);; Hôpital Ambroise Paré, Boulogne-Billancourt, France (F. El Sayed, A. Gruber, T. Bauer, B. Heym, C. Dupont, J.-L. Gaillard, M. Rottman);; Atelier de Bioinformatique, Paris, France (G. Sapriel);; Institut Pasteur, Paris (N. Fawal, S. Le Hello);; Hôpital Raymond Poincaré, Garches, France (M. Rottman)

**Keywords:** *Salmonella enterica* serotype Dublin, bone and joint infection, adaptation, epidemiology, bacteria, France

## Abstract

Genome degradation has been central to the adaptation of *Salmonella enterica* serotypes to their hosts throughout evolution. We witnessed the patho-adaptation of a strain of *Salmonella* Dublin (a cattle-adapted serotype) to a human host during the course of a recurrent prosthetic hip joint infection evolving over several years.

*Salmonella enterica* serotype Dublin is a host-adapted bacterium with cattle as a predominant reservoir and is responsible for invasive, potentially life-threatening infections in humans (*1**,**2*). In France, the epidemiology of *Salmonella* Dublin infections among humans corroborates surveillance data from the United States (*1*). *Salmonella* Dublin causes substantially more bloodstream infections and hospitalizations than other *Salmonella* serotypes. It is also much more likely to be isolated in metastatic foci of infection secondary to bacteremia. The prevalence of prosthetic joints in *Salmonella* Dublin patients is significantly greater than for other *Salmonella* cases (Table).

**Table:** Demographic characteristics of patients and sources for *Salmonella enterica* serotype Dublin and other *Salmonella* isolates, France, 2010–2016

Patient characteristic	*Salmonella* Dublin, no (%)	Other *Salmonella*, no. (%)	p value*
All cases, N = 63,264	642	62,622	
Sex†			
F	266	30,762	<0.01
M	363	30,401	
Age group, y‡			
<1	4 (0.6)	3,767 (6.0)	<0.01
1–5	33 (5.1)	15,302 (24.4)	<0.01
6–14	19 (3.0)	8,636 (13.8)	<0.01
15–64	199 (31.0)	23,653 (37.8)	NS
>65	375 (58.4)	9,801 (15.7)	<0.01
Unknown	12 (1.9)	1463 (2.3)	NS
Type of human sample§¶			
Feces	218 (34.0)	55,824 (89.1)	<0.01
Blood	264 (41.1)	3,268 (5.2)	<0.01
Urine	72 (11.2)	2,257 (3.6)	<0.01
Articular	20 (3.1)	30 (0.05)	<0.01
Pus	11 (1.7)	65 (0.1)	<0.01
Bile	1 (0.2)	32 (<0.1)	NS
Cerebrospinal fluid	0	23 (<0.1)	NS
Other	56 (8.7)	1123 (1.8)	<0.01
Hospitalization	368 (57.3)	20,556 (32.8)	<0.01

*Pearson χ^2^ test used for statistical comparisons with significant difference if p<0.05. NS, nonsignificant. †Numbers do not add to total because of patients for whom sex was unknown. ‡Median ages: *Salmonella* Dublin, 69.7 y (range <1–99); other *Salmonella*, 29.5 y (range <1–103); p<0.01. §Ratio of blood to 1,000 fecal isolates: *Salmonella* Dublin, 1,211; other *Salmonella*, 58; p<0.01. ¶Prosthetic joint prevalence among patients: *Salmonella* Dublin, n = 10/642 (1.5%); other *Salmonella*, 11/62,622 (<0.1%); p<0.01.

Host adaptation is central to pathogen evolution and is associated with gene acquisition, genome degradation (gene inactivation or deletion), or both. Genome degradation has played a major role in the adaptation of *S. enterica* serotypes Typhi and Paratyphi A to the human host throughout evolution (*3*). Degradation also has been recently reported in a severely immunocompromised patient in whom recurrent systemic *Salmonella* Enteritidis infections developed over 15 years (*4*). We report the phenotypic and genomic changes undergone by *Salmonella* Dublin throughout a recurrent prosthetic hip infection in an immunocompetent patient.

## The Study

In May 2011, a 74-year-old woman with bilateral hip prostheses (implanted in 1998 [right] and 2001 [left]) was admitted to intensive care for sepsis and left hip pain (Technical Appendix Figure). Blood cultures and a joint aspiration yielded pure cultures of *Salmonella* spp. She underwent debridement and implant retention surgery, followed by a 6-week intravenous course of cefotaxime and ciprofloxacin. Three years later, she sought care at the emergency department with acute-onset fever and prosthesis joint infection of the right hip and underwent right hip debridement and implant retention surgery. Blood cultures, joint aspirates, and all interoperative periprosthetic tissue samples yielded *Salmonella* spp. The patient received 2 weeks of intravenous amoxicillin and oral ofloxacin, was discharged, and received oral antimicrobial drugs for 10 more weeks. Six weeks after surgery, the surgical wound was healed, and the patient walked normally. One year later (2015), her primary care physician referred her to the hospital because of night fevers without local signs or implant dysfunction. Radioleucoscintigraphy showed right hip inflammation. Bilateral hip biopsies were performed, and the right hip biopsy sample tested positive for *Salmonella* spp. A right hip 1-stage exchange procedure was performed. All intraoperative periprosthetic tissue samples yielded *Salmonella* spp. A 6-week course of intravenous therapy with ceftriaxone and ciprofloxacin was administered. One year later (2016), the patient appeared to be free from infection and walked normally.

We characterized isolates by matrix-assisted laser desorption/ionization time-of-flight mass spectrometry (Bruker Daltonik GmbH, Bremen, Germany) and serotyping (French National Reference Center for *Salmonella*). We assessed carbohydrate metabolic activity using the API50CH system (bioMérieux, Marcy-l’Etoile, France) and biofilm formation using crystal violet (*5*). Antimicrobial susceptibility testing was performed using 2015 EUCAST (European Committee on Antimicrobial Susceptibility Testing) guidelines (http://www.eucast.org/clinical_breakpoints/). We conducted high-throughput whole-genome sequencing using the Illumina NextSeq 500 system (Illumina, San Diego, CA, USA). For each isolate, the paired-end reads were aligned against 2 *Salmonella* Dublin CT_020221853 and 3246 reference genomes to increase the single-nucleotide polymorphism (SNP) detection (GenBank accession nos. CP001144.1 and CM001151.1, respectively) (*6*) (online Technical Appendix). We annotated putative coding sequences using GeneMark (*7*) and performed ortholog identification, based on the prototypal human reference genome CT_020221853, using BLAST+ (*8*) with a 1 × 10^-10^ E-value threshold and confirmed by a synteny conservation check. We first identified candidate pseudogenized sequences using a proteome/proteome BLASTp approach (https://blast.ncbi.nlm.nih.gov/Blast.cgi): protein sequences of strains Str.2011, Str2014, and Str2015 with Query Coverage <80% relative to the reference strain orthologs were selected as potentially pseudogenized sequences. We then analyzed nucleic acid level sequences corresponding to the previously selected candidates to confirm the change in coding sequences, and to precisely determine the genetic event kind of nucleic acid change involved (frame-shift mutation or indel).

We studied 3 patient strains: Str.2011 (May 2011, left hip intraoperative periprosthetic tissue [HIPT]), Str.2014 (April 2014, right HIPT), and Str.2015 (June 2015, right HIPT). Unlike Str.2011, Str.2014 and Str.2015 were nonmotile with no detectable H antigen and had lost the ability to use 9 of the 18 carbohydrates used by Str.2011, with slight profile variations (Figure). All strains could form biofilms and had identical wild-type antimicrobial drug susceptibility profiles and unchanged MICs for β-lactams and quinolones, without mutations in *gyrA*, *gyrB*, *parC*, and *parE* genes. All 3 strains belonged to the sequence type 10 *Salmonella* Dublin population (http://mlst.warwick.ac.uk/mlst/dbs/Senterica). The mapping of Str.2011, Str.2014, and Str.2015 genomes against the *Salmonella* Dublin CT_020221853 and str.3246 reference genomes detected 451 and 268 SNPs, respectively, whereas the 3 patient strains differed by only 6 SNPs, strongly suggesting they derived from a single infecting strain. Four SNPs were nonsynonymous in coding genes; 2 SNPs were in an intergenic region (Figure).

**Figure:**
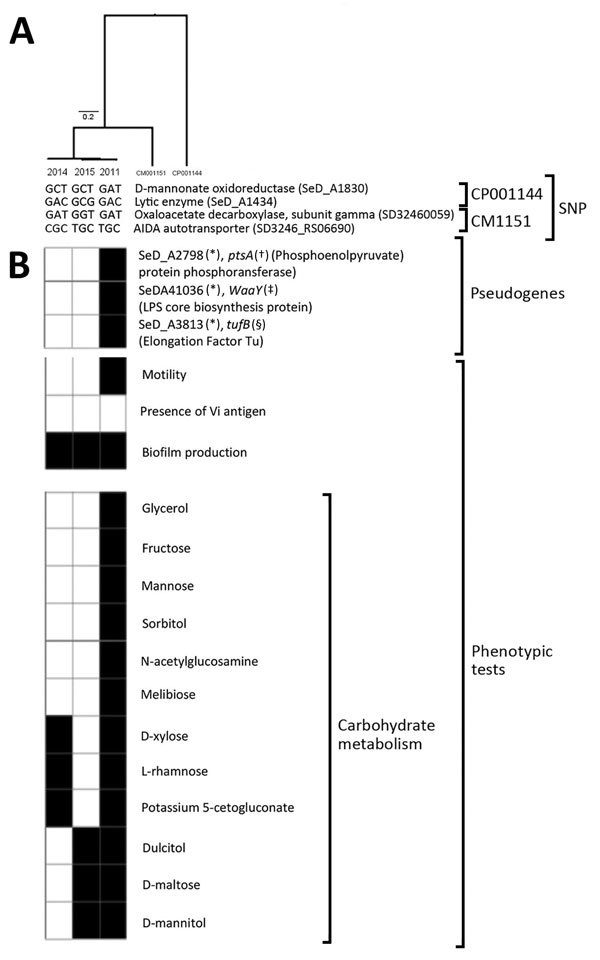
Genomic and phenotypic characteristics of *Salmonella enterica* serotype Dublin isolates Str.2011, Str.2014, and Str.2015 from a 74-year-old woman who had recurrent infections involving hip joint prostheses, France. A) Tree constructed after mapping the sequences of the isolates to reference genomes of *Salmonella* Dublin strains CT_020221853 and 3246 (GenBank accession nos. CP001144.1 and CM001151.1, respectively). The 4 nonsynonymous SNPs and the corresponding coding genes are shown. Scale bar indicates genetic distance. B) Genes in Str.2011 and altered in Str.2014 and Str.2015 (reference strain *Salmonella* Dublin CT_02021853) are indicated by black and white boxes, respectively. Positive phenotypic tests are indicated as black boxes. For carbohydrate metabolism, only carbohydrates used by Str.2011 and not used by Str.2014 and/or Str.2015 are shown. *Gene ID annotation based on reference strain *Salmonella* Dublin str. CT_02021853 (GenBank accession no. CP001144.1). Genetic events found: †14-bp deletion; ‡16-bp insertion; §790-bp deletion (total gene deletion). SNP, single-nucleotide polymorphism.

Comparative proteome/proteome analysis detected putative pseudogenized sequences that were due to indels within 3 coding sequences in Str.2014 and Str.2015. These pseudogenes were involved in carbohydrate transport (*ptsA*) (14-bp deletion), lipopolysaccharide (LPS) biosynthesis (*waaY*) (16-bp insertion), and protein synthesis (*tufB*) (790-bp deletion, total gene deletion); we identified no pseudogene among the genes of the flagellar regulon. The pseudogene involved in carbohydrate transport, *ptsA*, controls the import of carbohydrates, such as mannose, fructose, and N-acetyl-glucosamine (*9*), 3 carbohydrates used by Str.2011 but not Str.2014 or Str.2015. Mutation of the LPS biosynthesis gene *waaY* in Str.2014 and Str.2015 had apparently no effect on the expression of the *Salmonella* Dublin O antigen but might have affected flagellar assembly and function, as described for *Salmonella* Typhimurium and *Escherichia coli* (*10**,**11*). The mutation of *tufB* in Str.2014 and Str.2015 leads to a one third decrease in the production of the translation elongation factor EF-Tu (the other two thirds is synthetized from *tufA*) (*12*).

The patient in this study had no detectable gallbladder or intestinal carriage, which has been associated with recurrent *Salmonella* infections (*13*). She had no apparent immunodeficiency, a well-documented risk factor for invasive *Salmonella* Dublin infection. *Salmonella* Dublin most likely persisted as a biofilm at the surface of the implants, leading to recurrence of chronic disease despite prolonged antimicrobial therapy until the ablation of the infected material (*14*). The genomic changes we observed involved carbohydrate metabolism and LPS biosynthesis, as similarly reported for *Salmonella* Enteritidis isolates recovered from the bloodstream of a severely immunocompromised patient, years after the initial infection (*4*). Changes in carbon source availability are known to affect virulence gene regulators. In the presence/absence of a specific carbon source, specific virulence genes can be turned on or switched off, enabling pathogens to adapt to their new niche (*9*). Because LPS and flagellin are potent triggers of the inflammatory response, LPS alterations and the loss of flagellin expression (*15*) might have limited the host’s innate immune response to *Salmonella* Dublin and facilitated its persistence at the implant interface.

## Conclusions

This clinical case and other recent reports of patients with chronic infections highlight the remarkable adaptability of pathogens to a new niche. More specifically, these results show that the process of patho-adaptation of *Salmonella* serotypes may be extremely rapid and relies on mechanisms of genomic reshaping reminiscent of those found during the evolution of this pathogen in contact with humans. Enhanced awareness is warranted for *Salmonella* Dublin, especially in the elderly bearing prostheses.

Technical Appendix High-throughput genome sequencing of *Salmonella enterica* serotype Dublin isolates from a 74-year-old woman who had recurrent infections involving hip joint prostheses, France.
