# Supplementary material for: In-Host Adaptation of Salmonella enterica Serotype Dublin during Prosthetic Hip Joint Infection
Source: Emerg Infect Dis. 2018 Dec;24(12):2360–3. doi: 10.3201/eid2412.180214 (PMC6256398; doi:10.3201/eid2412.180214)
Supplement: Technical Appendix — High-throughput genome sequencing of Salmonella enterica serotype Dublin isolates from a 74-year-old woman who had recurrent infections involving hip joint prostheses, France. [file 18-0214-Techapp-s1.pdf]

Article DOI: https://doi.org/10.3201/eid2412.180214

In-Host Adaptation of *Salmonella enterica* Serotype Dublin during Prosthetic Hip Joint Infection

Technical Appendix

High-Throughput Genome Sequencing

High-throughput whole-genome sequencing was conducted on strains at the Plateforme de Microbiologie Mutualisée from the Pasteur International Bioresources network (Institut Pasteur, Paris, France). DNA extraction was carried out by using the MagNAPure 96 system (Roche). The libraries were prepared using the Nextera XT kit (Illumina) and the sequencing was done with the NextSeq 500 system (Illumina) generating 100–146-bp paired-end reads, yielding a mean of 61-fold coverage (minimum 32-fold, maximum 101-fold). Reads were trimmed and filtered using AlienTrimmer (*1*) with a quality Phred score threshold of 28 on a minimum length of 70 nt. De novo assembly was performed with the SPAdes V.3.6.0 assembler (*2*). For each isolate, the paired-end reads were aligned against 2 *Salmonella enterica* serotype Dublin CT_020221853 and 3246 reference genomes, to increase the single-nucleotide polymorphism (SNP) detection (GenBank accession nos. CP001144.1 and CM001151.1, respectively) using Bowtie2 with default parameters (*3*). SAMtools (*4*) were then used to build a genome index and identify SNPs from the Bowtie alignments. Several criteria were used to filter resulting SNPs: a minimum coverage (number of reads mapped to the reference genome) of 20 and a minimum quality score of each SNP at 25. For each approach, the resulting SNPs were concatenated to generate a filtered multiple alignment. The resulting sequences were further filtered to remove all SNPs present in insertion sequences identified by ISfinder (*5*). Other repetitive regions were identified by a self-self-BLAST analysis (*6*) of the reference sequence, using the following parameters: megablast (word size 28), identity percentage >95% and match length >400 bp. Finally, clusters of SNPs introduced by horizontal sequence transfer were detected and removed with Gubbins (*7*). Alignment was used as input for the construction of a phylogenetic tree using

MEGA6 using a maximum-likelihood approach (*8*). MEGA6 was ran using the general time reversible model and a Gamma distribution to model site-specific rate variation (i.e., the GTR+Γ substitution model). Hundred bootstrap replicate analyses were performed to assess maximum-likelihood phylogeny. The final tree was visualized in FigTree version 1.4.2 (http://tree.bio.ed.ac.uk/software/figtree/).

Availability of data

Genomics sequence assemblies used in this project are available online on the NCBI network under accession PRJNA433355 (available at http://www.ncbi.nlm.nih.gov/bioproject/433355).

References

1. Criscuolo A, Brisse S. AlienTrimmer removes adapter oligonucleotides with high sensitivity in short-insert paired-end reads. Commentary on Turner (2014) Assessment of insert sizes and adapter content in FASTQ data from NexteraXT libraries. Front Genet. 2014;5:130. PubMed http://dx.doi.org/10.3389/fgene.2014.00130

2. Bankevich A, Nurk S, Antipov D, Gurevich AA, Dvorkin M, Kulikov AS, et al. SPAdes: a new genome assembly algorithm and its applications to single-cell sequencing. J Comput Biol. 2012;19:455–77. PubMed http://dx.doi.org/10.1089/cmb.2012.0021

3. Langmead B, Salzberg SL. Fast gapped-read alignment with Bowtie 2. Nat Methods. 2012;9:357–9. PubMed http://dx.doi.org/10.1038/nmeth.1923

4. Li H, Handsaker B, Wysoker A, Fennell T, Ruan J, Homer N, et al.; 1000 Genome Project Data Processing Subgroup. The Sequence Alignment/Map format and SAMtools. Bioinformatics. 2009;25:2078–9. PubMed http://dx.doi.org/10.1093/bioinformatics/btp352

5. Siguier P, Perochon J, Lestrade L, Mahillon J, Chandler M. ISfinder: the reference centre for bacterial insertion sequences. Nucleic Acids Res. 2006;34:D32–6. PubMed http://dx.doi.org/10.1093/nar/gkj014

6. Altschul SF, Gish W, Miller W, Myers EW, Lipman DJ. Basic local alignment search tool. J Mol Biol. 1990;215:403–10. PubMed http://dx.doi.org/10.1016/S0022-2836(05)80360-2

7. Croucher NJ, Page AJ, Connor TR, Delaney AJ, Keane JA, Bentley SD, et al. Rapid phylogenetic analysis of large samples of recombinant bacterial whole genome sequences using Gubbins. Nucleic Acids Res. 2015;43:e15. PubMed http://dx.doi.org/10.1093/nar/gku1196

8. Tamura K, Stecher G, Peterson D, Filipski A, Kumar S. MEGA6: molecular evolutionary genetics analysis version 6.0. Mol Biol Evol. 2013;30:2725–9. PubMed http://dx.doi.org/10.1093/molbev/mst197

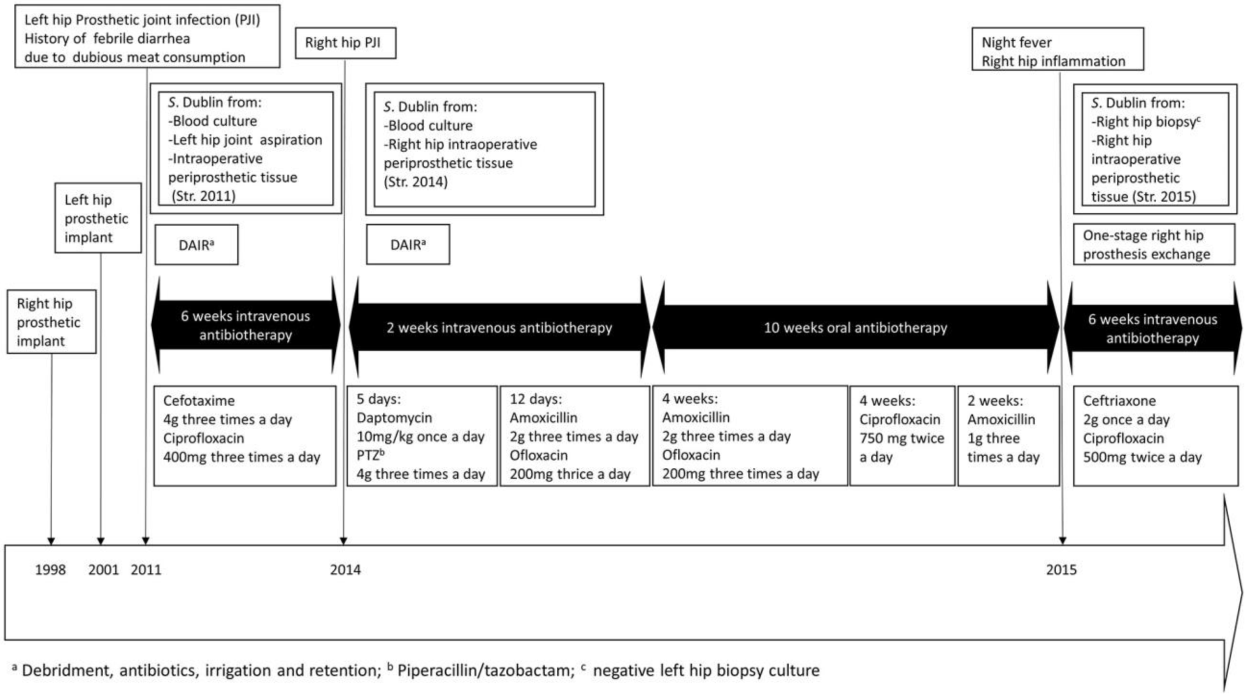

Technical Appendix Figure. Clinical case summary for immunocompetent patient who acquired *Salmonella* Dublin infection after receiving a prosthetic hip joint.
